# UPΦ phages, a new group of filamentous phages found in several members of *Enterobacteriales*

**DOI:** 10.1093/ve/veaa030

**Published:** 2020-06-22

**Authors:** Jason W Shapiro, Catherine Putonti

**Affiliations:** v1 Department of Biology, Loyola University Chicago, 1032 W Sheridan Rd, Chicago, IL 60660, USA; v2 Department of Computer Science, Loyola University Chicago, 1052 W Loyola Ave, Chicago, IL, 60626, USA; v3 Bioinformatics Program, Loyola University Chicago, 1052 W Loyola Ave, Chicago, IL 60626, USA; v4 Department of Microbiology and Immunology, Stritch School of Medicine, Loyola University Chicago, 2160 S First Ave, Maywood, IL 60153, USA

**Keywords:** bacteriophage, inovirus, prophage, bladder

## Abstract

Filamentous phages establish chronic infections in their bacterial hosts, and new phages are secreted by infected bacteria for multiple generations, typically without causing host death. Often, these viruses integrate in their host’s genome by co-opting the host’s XerCD recombinase system. In several cases, these viruses also encode genes that increase bacterial virulence in plants and animals. Here, we describe a new filamentous phage, UPϕ901, which we originally found integrated in a clinical isolate of *Escherichia coli* from urine. UPϕ901 and closely related phages can be found in published genomes of over 200 other bacteria, including strains of *Citrobacter koseri*, *Salmonella enterica*, *Yersinia enterocolitica*, and *Klebsiella pneumoniae*. Its closest relatives are consistently found in urine or in the blood and feces of patients with urinary tract infections. More distant relatives can be found in isolates from other environments, including sewage, water, soil, and contaminated food. Each of these phages, which we collectively call ‘UPϕ viruses’, also harbors two or more novel genes of unknown function.

## 1. Introduction

Phages are often described by their potential to kill their hosts. Obligately lytic phages kill their hosts following infection, whereas temperate phages may lie dormant as prophages within lysogenized bacteria for several generations before entering a lytic cycle. While killing by phages has immediate consequences for bacterial ecology and has led to the revival of phage therapy for treating bacterial infections (Kortright et al. 2019), many phages also carry genes that alter bacterial behavior (Mai-Prochnow et al. 2015; Warwick-Dugdale et al. 2019). The effects of these phage-encoded genes range from modifying photosynthesis in cyanobacteria (Sieradzki et al. 2019) to producing toxins in potential pathogens (Waldor and Mekalanos 1996).

Filamentous phages in the family *Inoviridae* go beyond the standard dichotomy of viral lysis and lysogeny. Instead, the majority of characterized inoviruses maintain productive infections over multiple bacterial generations, without killing their hosts. In many cases, these phages integrate as tandem repeats (e.g. Derbise et al. 2007) into their hosts’ genomes at a locus called the *dif* site (Mai-Prochnow et al. 2015). The *dif* site is a 28 bp region at the bacterial terminus that includes two short palindromic regions recognized separately by the site-specific recombinase pair XerC and XerD. In bacteria with this system, XerCD is responsible for resolving chromosome dimers during DNA replication and cell division (Carnoy and Roten 2009). Filamentous phages co-opt this system by carrying their own copy of the *dif* site within their genomes, causing XerCD to confuse phage DNA for bacterial DNA. After integration, new phages are produced at relatively low rates and may be maintained indefinitely within the bacterial population (Val et al. 2005). Other filamentous phages that lack a *dif* site can also integrate elsewhere in the bacterial genome by encoding their own integrases, as in the Pf bacteriophages of *Pseudomonas aeruginosa* (Mai-Prochnow et al. 2015).

Filamentous phages can have dramatic effects on their hosts’ phenotypes. In *Vibrio cholerae*, the phage CTXϕ encodes the toxin genes responsible for bacterial virulence in humans (Waldor and Mekalanos 1996). Similarly, filamentous phages have been associated with the virulence of other human pathogens including hemorrhagic *Escherichia coli*, *Yersinia pestis* (the cause of plague), and *P. aeruginosa* in both patients with cystic fibrosis and skin wounds (Gonzalez et al. 2002; Derbise et al. 2007; Sweere et al. 2019). Inoviruses have also been correlated with the increased virulence of agricultural pests like tomato wilt and potato blight (Kamiunten and Wakimoto 1982; Yamada 2013). Across these various infections, filamentous phages may carry toxin genes, alter bacterial motility (Jian, Xiao, and Wang 2013) and biofilm formation (Rice et al. 2009; May, Tsuruta, and Okabe 2011), or indirectly interfere with the immune system’s ability to clear the bacterial infection (Sweere et al. 2019). Despite their myriad effects on a wide range of clinically and agriculturally important hosts, these phages remain understudied. As of this writing, there are only forty-six recognized members of *Inoviridae* in RefSeq, and only thirty-three with established taxonomy by the International Committee on Taxonomy of Viruses (ICTV). Nonetheless, recent work has shown that thousands of potential inoviruses can be found as prophages in published bacterial genomes and metagenomes (Roux et al. 2019).

Here, we describe a novel filamentous phage, UPϕ901, discovered as a prophage in a clinical *E. coli* isolate from a patient urine sample. UPϕ901 is most closely related to the non-integrating phages I2-2 and IKe in the *Lineavirus* genus of the *Inoviridae.* Like many filamentous phages, UPϕ901 integrates as a tandem repeat at the *dif* site in its hosts. Using the phage sequence from our isolate as a query, we searched for close relatives of UPϕ901 in published bacterial genomes and assemblies. We found homologous prophages in over 200 strains of *E. coli*, *Citrobacter koseri*, *Klebsiella pneumoniae*, *Salmonella enterica*, and *Yersinia enterocolitica.* The most similar phages tended to come from patient urine samples or from the blood or feces of patients with urinary tract infection (UTI); more distant relatives could be found in soil, water, animal feces, and contaminated food. We refer to the collection of related filamentous phages as ‘UPϕ viruses’. In several cases, identical UPϕ strains were found infecting multiple bacterial genera, suggesting recent host switching events. Last, we describe the prevalence of putative UPϕ viruses in metagenomes.

## 2. Methods

### 2.1 Bacterial *s*trains

Clinical isolates of *E. coli* and *C. koseri* (see Table 1) were provided by the Wolfe Lab at Loyola University Chicago and were originally isolated as part of separate IRB-approved studies on the urinary microbiota of women with and without symptoms of UTIs or other urinary ailments (Price et al. 2016a,b; Garretto 2019; Penckofer et al. 2020). These strains and their GenBank accessions (where available) are summarized in Table 1. *Citrobacter koseri* strains in this study have not been fully sequenced. *Escherichia coli* JE-1 is an IncI plasmid-bearing strain typically used to propagate phage I2-2 and was obtained from the Felix d’Herelle Reference Center for Bacterial Viruses (Université Laval, QC, Canada).

**Table 1.** Bacterial strains in this study.

Strain ID	Species	Has UPϕ?	WGS accession
UMB0731	*E. coli*	No	NZ_RRWP00000000^a^
UMB0901	*E. coli*	Yes	NZ_PKHH00000000
UMB1220	*E. coli*	Yes	NZ_RRVZ00000000
UMB1526	*E. coli*	Yes	NZ_RRVH00000000
UMB5814	*E. coli*	Yes	NZ_RRUX00000000
UMB6655	*E. coli*	No	NZ_RRUL00000000
UMB6890	*E. coli*	No	NZ_RRUI00000000
UMB1389	*C. koseri*	No	Unsequenced^b^
UMB7451	*C. koseri*	Yes	Unsequenced
UMB8248	*C. koseri*	No	Unsequenced
JE-1	*E. coli*	No	Unavailable

^a^ UMB0901 was sequenced as ‘E75’ in its original BioProject (PRJNA316969).

^b^ *Citrobacter* strains not sequenced at time of writing. Phage identified by PCR.

### 2.2 Identifying phage in bacterial genomes

UPϕ901 was originally discovered as part of predicting phage sequences using PHASTER (Arndt et al. 2016) in UMB0901 and other *E. coli* isolates in a previous study (Miller-Ensminger et al. 2018; Garretto 2019). (The ‘UP’ in its name originated uncreatively from ‘urine project’, though not all related viruses are necessarily found in urine, as described in Section 3.) To identify UPϕ901 in other published genomes, we took advantage of its integration at its hosts’ *dif* sites as a tandem repeat. We defined a preliminary BLAST (Altschul et al. 1990) query sequence for UPϕ901 as the region starting with the *dif* locus in UMB0901 and extending to the first phage repeat (7,560 bp). Throughout this paper, we will refer to the specific sequence (and homologs that are over 99% identical by amino acid sequence) as ‘UPϕ901’. Remaining phages that share a common set of core genes (identified below) and with less than 99 per cent amino acid identity are collectively referred to as ‘UPϕ viruses’. The *dif* site itself is not repeated in full within the tandem duplications. We then used this single copy of the phage as a query for a blastn search using the NCBI web tool (https://blast.ncbi.nlm.nih.gov) with default parameters. This BLAST search identified prophages with over 99 per cent query coverage and over 98 per cent nucleotide identity to UPϕ901 in strains of *C. koseri*, *E. coli*, *K. pneumoniae*, *S. enterica*, and *Y. enterocolitica*. Notably, neither PHASTER nor VirSorter (Roux et al. 2015) consistently predicted UPϕ901 homologs in bacteria that had significant BLAST hits. This high false-negative rate for these two tools is likely due to UPϕ901’s short genome and the reliance of these tools on information about known viruses. We have not tested the newer tool Inovirus_detector (Roux et al. 2019) with genomes carrying UPϕ901 homologs.

We next downloaded all available draft and complete assemblies on NCBI for the five bacterial species listed above (as of August 2018). Each assembly was queried locally with tblastx with default parameters to identify contigs containing putative phage regions. We retained any contigs containing a hit covering at least 1,000 nucleotides of the UPϕ901 query with over 75 per cent nucleotide identity. In no case did we observe a putative UPϕ901 homolog on two contigs in the same assembly. The start positions of each phage were then confirmed by a separate local blastn search for the *dif* site (as identified in Carnoy and Roten 2009), and stop positions were identified by confirming the first repeat in the BLAST results. These repeats typically matched just the first fifteen bases in the *dif* site, and we accepted putative hits that covered at least fourteen of the twenty-eight *dif* site nucleotides with at least 85 per cent identity. Due to varying assembly quality, not all assemblies included phage repeats, and some sequences did not include a *dif* repeat as an obvious stopping point. In these instances, we could only rely on the end position of the last significant hit in the initial tblastx query.

To confirm the sensitivity of our UPϕ901 detection, we then blasted each of the 229 putative UPϕ901-infected genomes against a query for the related phage, Ypfϕ (from *Y. pestis* CO92, GenBank: CP009973), which has been found at *dif* sites in other studies (Derbise et al. 2007). We liberally accepted all putative hits, regardless of identity, and found a single case (GCA_001519645) with a cumulative query coverage over 1,000 bp. We queried both UPϕ901 and Ypfϕ in SRA BLAST searches against the original reads for this assembly (SRX1528813).

### 2.3 Phylogenetic analyses

We used Anvi’o (Eren et al. 2015) to facilitate gene clustering and annotation for the putative phage regions found in GenBank assemblies. Standard annotation tools fail to identify inovirus genes accurately, so we then used blastp through the NCBI web tool with individual phage IKe (GenBank: NC_002014) and I2-2 (GenBank: NC_001332) genes to confirm each gene’s correct annotation. These were identified by visually comparing the aligned genes rather than relying on a significance threshold, as homologous sequences may share less than 30 per cent amino acid identity in these phages. Full-length UPϕ901 and most of the putative phage regions have every gene found in IKe and I2-2 (as in Fig. 2).

We identified nine UPϕ901 core genes to use for phylogenetic inference (genes *I*, *II*, *III*, *IV*, *V*, *VI*, *VIII*, *h1*, and *h2*). Genes *VII* and *IX* were excluded from analyses, because they consist of only about thirty amino acids and were not consistently identified as genes. Gene *X* was also excluded as it is contained entirely within gene *II*. Next, we used MAFFT (Katoh and Standley 2013) to align each of the core genes. In reviewing individual gene alignments, we identified cases in genes *I*, *II*, *III*, and *IV* where the original gene calls by Anvi’o split the open reading frame (ORF) into two pieces because of early stop codons. It is unknown if these genes are truly pseudogenized or if they might still encode functional proteins. For the purpose of building the trees, we concatenated the two halves of these genes, since their complete sequences reflect evolutionary relationships, even if they might not be translated in full. Both the original and concatenated versions of these sequences are provided in the data repository for this work.

We then built a phylogeny of the 229 complete prophages with IQ-TREE version 1.6.12 (Nguyen et al. 2015) using the concatenated alignment of each core UPϕ901 gene amino acid sequence with 1,000 bootstraps. We took advantage of IQ-TREE’s integrated ModelFinder (Kalyaanamoorthy et al. 2017) option to perform model selection. This step identified VT+F+R2 (variable time with empirical state frequencies and two rate categories) as the best substitution model according to Bayesian Information Criterion (BIC). Individual gene trees shown in Supplementary Fig. S1 were built using FastTree (Price, Dehal, and Arkin 2010).

We also generated a bacterial phylogeny for the phage hosts, using Anvi’o to identify a set of 400 single-copy core genes shared among each host that also contained a prophage in the virus phylogeny. These genes were aligned within Anvi’o using MUSCLE (Edgar 2004) and concatenated. We used IQ-TREE as above to build the final phylogeny. For the host tree, ModelFinder identified JTT+F+R10 (Jones–Taylor–Thornton (Jones, Taylor, and Thornton 1992) with empirical state frequencies and ten rate categories) as the best substitution model by BIC.

Trees were visualized using iTOL (Letunic and Bork 2019) and the ape package (Paradis and Schliep 2019) in R (R Core Team 2013). Additional metadata for tree visualization were obtained from NCBI, EBI, or from the literature. Key metadata included the type of material sampled for isolating bacteria (e.g. soil, blood, urine, feces) and the more general source of that material (e.g. environmental, animal, human, food). The full metadata associated with each sample is provided in the data repository (see Data availability). Nine strains had no available metadata from a publication or database. Twenty-two other strains without available metadata were from the ‘100K Pathogen Genomes Project’ (BioProject PRJNA186441; Weimer 2017) and are identified as ‘100K Project’ in figures.

### 2.4 Phage in SRA metagenomes

We used searchsra (Levi et al. 2018; www.searchsra.org) to check for UPϕ901 relatives in metagenomes. We used a truncated 6,703 bp UPϕ901 reference that included only the core UPϕ virus genes used in phylogenetic analyses. The results from the search contained over 100,000 potential hits, most of which are false positives returned from a small number of erroneous reads aligning to the reference genome. Following the searchsra authors’ GitHub repository (https://github.com/linsalrob/SearchSRA), we first identified the depth of coverage across our reference genome for each potential hit. We used the pileup.sh function from BBMap (Bushnell 2014) to perform this step. We filtered results by both the query coverage and smoothness of coverage (Aziz et al. 2015) to identify true positives. A detailed description of these filtering steps with examples of true and false-positive coverage plots is provided in the Supplementary Methods. Metadata for the likeliest true-positive results are available in Supplementary File S1 and as part of the data repository. Statistical analysis related to these metadata was done in R.

### 2.5 Phage presence in culture medium

UPϕ901-infected strains were grown in lysogeny broth (LB) at 37 °C with moderate shaking overnight. The following morning, 1 ml of each culture was removed, centrifuged at 16,000 g for 1 min, and the supernatants were filtered through 0.2-μm cellulose acetate syringe filters. An 80 μl sample of each filtrate was then treated with OPTIZYME DNase I (Fisher BioReagents BP81071) for 30 min, followed by heat inactivation with EDTA at 65 °C for 10 min. This DNase step was included to remove any bacterial genomic DNA from the supernatant, which could result from cell death due to lysis from other prophages or shearing forces. DNase-treated samples were incubated at 95 °C for 10 min to denature phage protein coats and expose the phage ssDNA. We then amplified phage genomic DNA using UPϕ901-specific primers, UPphi_shortFW (GGGTTTATCAGAGGGGTCAG) and UPphi_shortRV (AGGATGGCTCTAAGTCAACG). 16S PCR (63 F/1387R primers) was used to confirm the absence of bacterial genomic DNA in the DNase-treated filtrates. The UPϕ901 and 16S PCRs were performed on 1 μl of unfiltered culture as positive controls. Positive UPϕ PCR products were verified by Sanger sequencing using the UPphi_shortFW primer.

### 2.6 Phage infection assays

We tested the potential for UPϕ901 to infect new hosts using standard plaque assays by spotting 5 μl of filtrate from UMB0901 (as described above) on 0.7 per cent agar LB overlays containing candidate bacterial hosts (UMB0731, UMB6655, UMB6890, JE-1).

We also tested for new infections using PCR. Colonies of prospective host strains were added to 5 ml of LB supplemented with 50 μl of filtered supernatant from UMB0901. Uninfected controls of each strain were also grown. These cultures were incubated for 18 h. Following growth, 2 μl of each culture were used in a PCR designed to test for integration into the new host’s chromosome. The forward primer (IntCheck_FW: GTGTGTGGATGTGAATGGTG) in the assay was based on a conserved sequence in all tested hosts just upstream of the dif site, and the reverse primer (IntCheck_RV: CTGGCAGAACGAACGATTAC) recognized a region early in the phage genome. This PCR can only amplify DNA if UPϕ901 integrates into a new genome. An overnight culture of UMB0901 was used as a positive control for each reaction, and the uninfected cultures were used as negative controls.

## 3. Results

### 3.1 Initial identification

**Figure 1.**
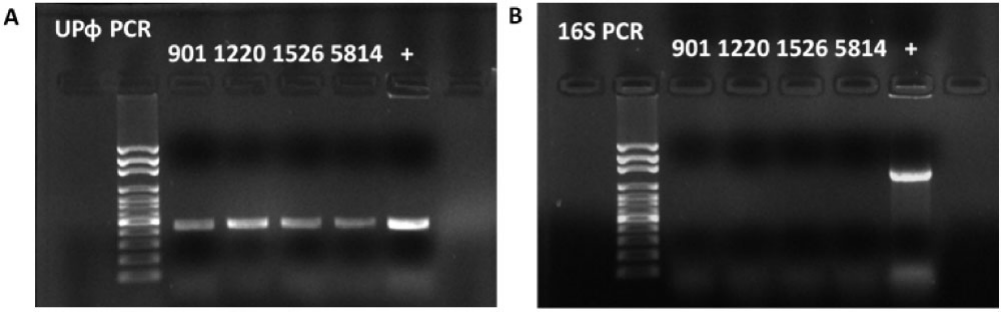
Phage DNA confirmation. PCR results using (A) UPϕ or (B) 16S primers after DNase treatment to remove bacterial genomic DNA from filtrates of UMB0901, UMB1220, UMB1526, and UMB5814 cultures. The + control lane was from UMB0901 colony PCR.

We originally identified UPϕ901 in a clinical isolate of *E. coli* (UMB0901) as part of separate work examining prophages present in the urinary microbiome (Garretto 2019). We then found prophages with over 99 per cent nucleotide identity in three other patient isolates in our lab collection (UMB1220, UMB1526, and UMB5814). In each case, we confirmed by PCR that the phage is shed from infected bacteria, as centrifuged culture supernatant treated with DNase is positive for UPϕ901 DNA (Fig. 1A) but negative for bacterial genomic DNA (Fig. 1B). Sanger sequencing confirmed that each PCR product was a true match to the UPϕ901 genome.

### 3.2 Comparison to characterized inoviruses

UPϕ901 is approximately 50 per cent identical to phages IKe and I2-2 (70% coverage and ∼70% amino acid identity for covered genes), each a non-integrating member of the *Lineavirus* genus of filamentous phages. These phages are closely related to the F-specific filamentous phage, M13. We also compared UPϕ901 to Ypfϕ, a *dif-*integrating filamentous phage that infects pathogenic strains of *E. coli* and *Y. pestis*. Overall, Ypfϕ shares less than 30 per cent amino acid identity with UPϕ901.

**Figure 2.**
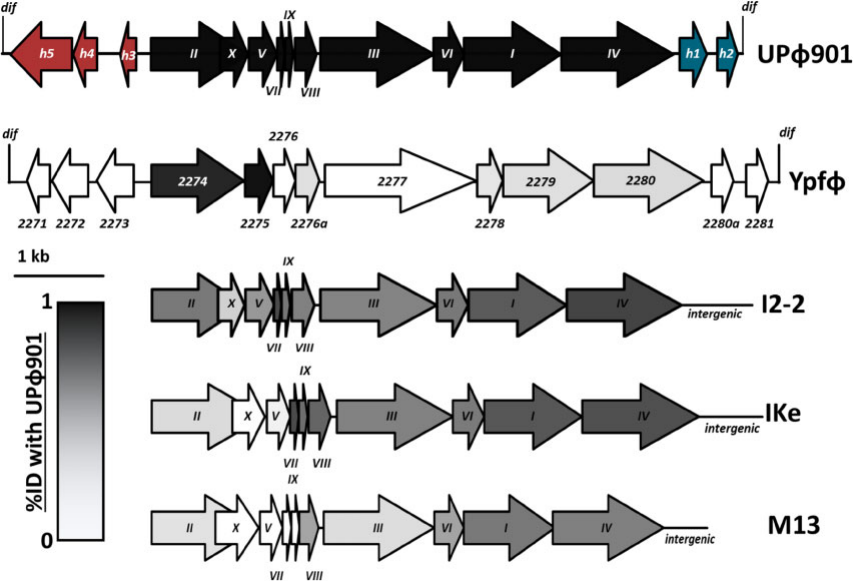
Comparison of UPϕ901 to related inoviruses. Roman numerals are the names for core inovirus genes common in M13 and the *Lineavirus* members. Ypfφ genes labeled as in Derbise *et al*. (2007). Genes shaded gray are darker if they are more similar to the sequence from the UPϕ901 prophage in UMB0901.

Figure 2 shows how UPϕ901’s genome compares with these other inoviruses, with each gene shaded according to its amino acid similarity with the homolog in UPϕ901. UPϕ901 contains each of the ‘core’ genes characteristic of filamentous coliphages with gene order preserved. UPϕ901 and Ypfϕ are less than 20 per cent identical in all genes except for genes *II* and *V*, which are each over 85 per cent identical between these phages. Genes *II* and *V* are both involved in regulating phage DNA replication, and this conserved module is less than 15 per cent similar in IKe and M13.

Two other differences set UPϕ901 apart from the non-integrating phages, I2-2, IKe, and M13: 1, UPϕ901 includes a copy of the *E. coli dif* site at the start of its genome, enabling it to integrate via the XerCD recombinase system; 2, UPϕ901 encodes two putative genes of unknown function within a region that is intergenic in I2-2, IKe, and M13. These genes are not homologous to any known protein, and all attempts to find annotated homologs of these genes in GenBank returned hits to hypothetical proteins in prophages related to UPϕ901. In addition to these hypothetical proteins, the UPϕ901 prophage region also contains three ORFs in reverse orientation just downstream of the *dif* site. This genome organization is mirrored by Ypfϕ and other integrating filamentous phages, including CTXϕ, where accessory genes often surround the core functions (Derbise et al. 2007; Mai-Prochnow et al. 2015).

### 3.3 Comparative genomics

UPϕ901 strains share nearly 100 per cent amino acid identity across infected clinical isolates of *E. coli* in our lab collection. The only distinction is that the attachment protein, g3p (encoded by gene *III*), in UPϕ901 has an additional repeat of a glycine-rich motif (‘GGGES’) than the viruses found in UMB1220, UMB1526, and UMB5814. As with many other filamentous phages, UPϕ901 is integrated as a tandem repeat at the *dif* site in each of these genomes. In the case of UMB0901, we re-assessed our own assembly by aligning the raw reads to the contig with UPϕ901 and estimating the depth of coverage (Supplementary Fig. S2). On average, the UPϕ901 prophage region has three times the coverage of the surrounding loci, indicating a tandem triple in UMB0901.

Using the UPϕ901 sequence as a BLAST query, we searched GenBank for homologous prophages in other bacteria. Prophages with over 98 per cent nucleotide identity across the UPϕ901 query sequence could be found in published genomes of *E. coli*, *S. enterica*, *C. koseri*, *K. pneumoniae*, and *Y. enterocolitica*. We then downloaded all available assemblies from GenBank of these five species (26,397 in total) and used tblastx to identify viruses integrated at *dif* sites. In all, 331 genomes harbored a potential UPϕ901 prophage (see Table 2 for a summary by host). Of these 331 putative phages, 229 contained each gene found in UPϕ901, excluding the three ORFs neighboring the *dif* site. The third of these ORFs (labeled *h3* in Fig. 2) is present in 221 of these 229 putative prophages, whereas *h4* and *h5* are found in only 56 and 57 genomes, respectively. For the remainder of this paper, we will refer to this collection of 229 strains as ‘UPϕ viruses’. The 102 remaining genomes contained a significant hit to UPϕ901, but assembly or sequencing quality was inadequate to predict full phage genomes reliably. We also checked each of the 229 UPϕ-infected genomes for Ypfϕ to rule out possible false-positive results. We found one instance (*E. coli* assembly GCA_001519645) of coinfection between a *dif*-integrated UPϕ prophage and a putative Ypfϕ prophage integrated at a different locus. None of the remaining 228 genomes harbored a prophage resembling Ypfϕ.

**Table 2.** Prevalence of UPϕ in GenBank assemblies.

Host species	GenBank assemblies	Assemblies with UPϕ hit
*E. coli*	12,401	189
*S. enterica*	9,144	95
*K. pneumoniae*	4,647	29
*Y. enterocolitica*	177	10
*C. koseri*	28	8

**Figure 3.**
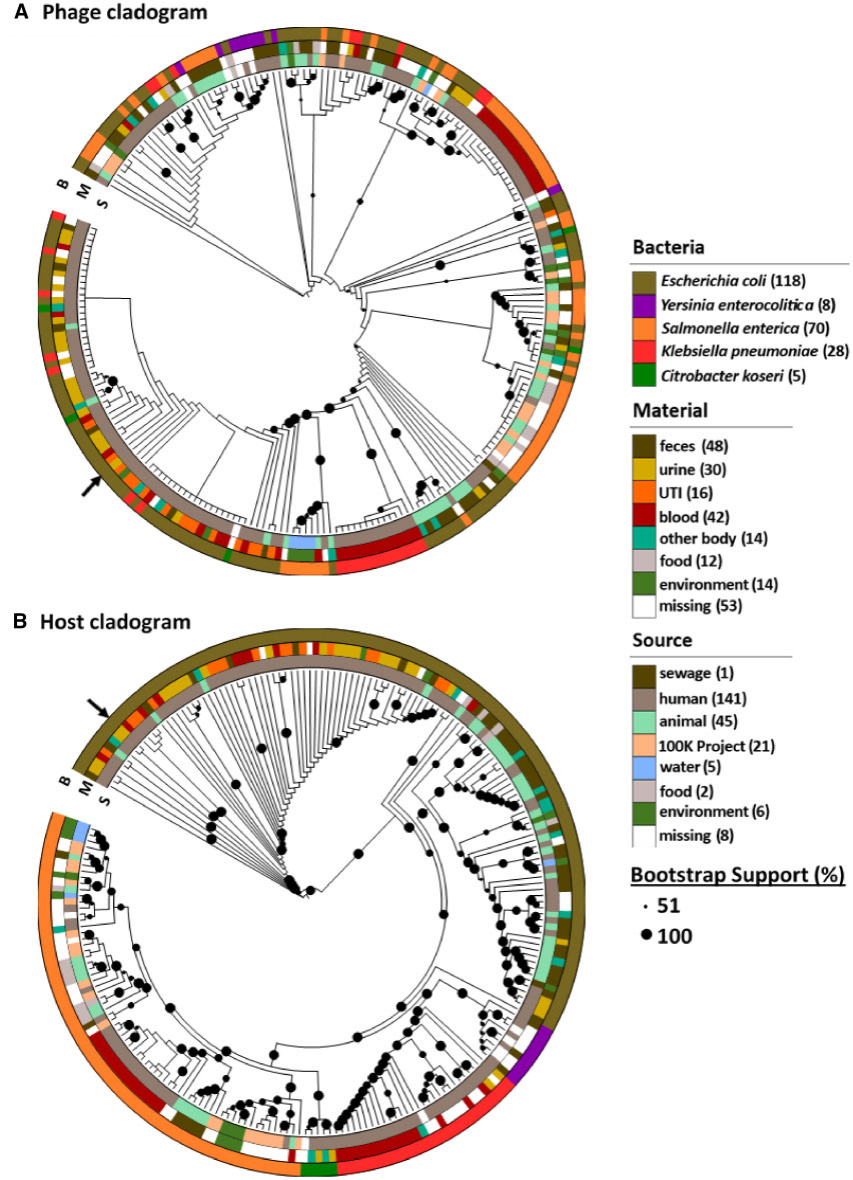
Cladograms of UPϕ prophages in GenBank assemblies (A) and their hosts (B). The outer ring is the host species, the middle ring is the sample material, and the inner ring is the source environment. Black arrows indicate (A) UPϕ901 and (B) UMB0901. Samples sizes shown in parentheses in the legend. Trees are based on amino acid sequences of core genes. Bootstrap support is shown by the size of black dots with at least 51 per cent support. High-resolution trees with scale bars and bootstrap supports are in Supplementary Fig. S3.

We then built a phylogeny for the 229 complete UPϕ prophages. Overall, the phylogeny reflects low genetic variation across the UPϕ viruses, and most clades are separated by short branches and include polytomies. Even the more distant phage relatives found in *Y. enterocolitica* are 98 per cent identical in amino acid sequence to the original UPϕ901 core genes. As a result, the tree has multiple splits with bootstrap support below 50 per cent. For this reason, we show the tree as a cladogram in Fig. 3A and will not attempt to over-interpret the tree topology. (A high-resolution phylogeny with complete bootstrap supports is shown in Supplementary Fig. S3.) For each of the genomes in the phylogeny, we identified metadata, where available, for the sample source material (e.g. urine, feces, blood) and source environment (e.g. human, animal, environmental) and added these data to the tree visualization.

UPϕ901 is indicated by an arrow in Fig. 3A and is part of a clade of sixty-five phages. Fifty-five of these prophages come from *E. coli*, of which thirty-six came from urine (including fourteen UTI samples). Two large polytomies make up the majority of this group, and at least one sample (assembly GCA_000457405) was from the blood of a patient with UTI-induced bacteremia. In addition, each polytomy includes cases where identical phages (i.e. 100% amino acid identity across the genes used in the phylogeny) were also found within *C. koseri* and *K. pneumoniae*. This group contains three samples from animals. The rest of the tree includes seventy prophages found in *S. enterica* (from both animals and humans), though phages infecting each of the other host species are also present. None of these *S. enterica* samples were isolated from urine, and only ten of the remaining ninety-four sequences came from urine or UTI samples. The total counts for each host species, sample material, and source are indicated in the legend for Fig. 3.

We next constructed a phylogeny for the host bacteria, using a set of conserved single-copy genes identified with Anvi’o (Eren et al. 2015). The bacterial tree shows the expected breakdown by host species, with additional structure corresponding to sample material and whether the strain was from human or animal sources. Branch supports are generally higher in the bacterial tree except for clades with very short branches. Due to the low branch support of the phage tree, it is difficult to compare the phage and host tree topologies.

**Figure 4.**
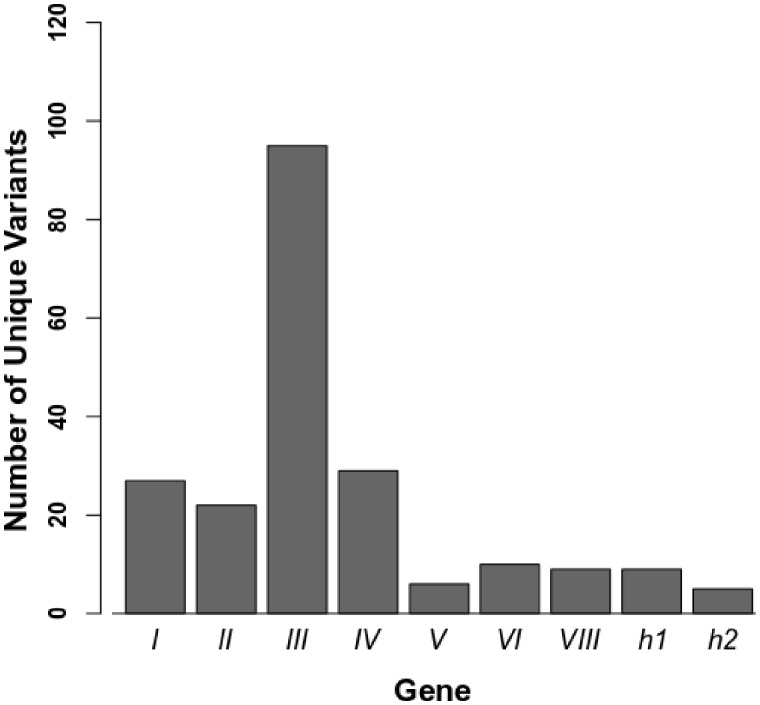
Number of unique amino acid sequence variants for each UPϕ gene used to build trees.

As expected from the short branches of the phage tree, individual phage gene trees (Supplementary Fig. S1) show little variation in amino acid sequence across the phages, with most genes composed of a small number of unique variants (Fig. 4). Gene *III* stands out as having the most variation, with ninety-five distinct amino acid sequences among the 229 genomes. Examining the alignment for gene *III*, most of the variation is in the number of glycine-rich repeats in the region that also varied in our lab isolates.

### 3.4 Phages in metagenomes

We next used searchsra (Levi et al. 2018) to find relatives of UPϕ901 in metagenomes. The initial results included over 100,000 sequencing runs with at least one read aligned to UPϕ901. After a series of filtering steps to remove erroneous data and false-positive results (see Supplementary Methods) we identified 257 SRA runs that were most likely to have true-positive results. We then used SRA BLAST (Camacho et al. 2009) to validate these putative hits and identified any instances of redundant BioSample numbers (multiple sequencing runs from the same sample) or redundant sample source (e.g. multiple samples from the same individual). This validation step identified 78 true positive, non-redundant samples that covered over 75 per cent of the UPϕ901 reference genome with over 90 per cent sequence identity. SRA BLAST is sensitive enough to distinguish between UPϕ and Ypfϕ prophages (Supplementary Fig. S4).

These positive metagenomic results include sixty-four from gut, five from urine, and four from environmental samples. Two additional hits came from reference isolates of *C. koseri* and from mock communities used in validating microbiome projects. (Full metadata are provided in Supplementary File S1.) Five of the positive gut samples were from non-human mammals, including three from cats and two from pandas. Environmental samples included two from the New York subway, one from wastewater, and one from river sediment. Most notably, twenty-four of the sixty-four unique gut samples came from nine different studies of the infant microbiome and included children from China, Estonia, Finland, Singapore, and the USA. One hypothesis for this high number of infant samples carrying UPϕ-infected bacteria is that UPϕ may be more common in hospital-acquired Proteobacteria.

Several filamentous phages have been associated with pathogen virulence (Mai-Prochnow et al. 2015), including one study identifying their potential involvement in cases of neonatal meningitis caused by *E. coli* O18:K1:H7 (Gonzalez et al. 2002). We were interested to see if any of the infant microbiome studies identified through searchsra might shed light on potential connections between UPϕ viruses and bacterial virulence.

In one study (Ward et al. 2016), the authors sequenced the gut microbiomes of 144 pre-term and twenty-two full-term infants and identified cases where necrotizing enterocolitis (NEC) was associated with strains of uropathogenic *E. coli*. Furthermore, the anonymized patient metadata were available with the publication and could be matched to sample metadata from the SRA. This patient data included indicator variables for high (over one or ten) per cent abundance of *E. coli* and *K. pneumoniae*, as well as information on patient outcomes. We identified nine patients in this study that carried bacteria infected by a putative UPϕ prophage (patient IDs: 11211, 11961, 11962, 12321, 21461, 30031, 30081, 30251, 30252). Of these, six contained *E. coli* and five contained *K. pneumoniae*; two patients had both bacteria present. The overall rate of UPϕ presence (9/166) is about four times higher than across GenBank assemblies of *E. coli* and *K. pneumoniae* (218/17048). This difference represents a significant enrichment (*χ*^2^ = 170.44, P < 0.001) in this study. Moreover, two of the nine patients were ultimately diagnosed with NEC. This is not significantly different (*χ*^2^ = 0.0011, P = 0.973) from the rate of NEC without UPϕ in the study (25/157). In addition, four of the patients (IDs starting with ‘30’) were full-term and not treated in the NICU. The rate of UPϕ prophages among the full-term infants (4/22) is significantly greater than among the pre-term patients (5/144) (*χ*^2^ = 5.44, P = 0.020). Thus, while it is possible that UPϕ is more common in hospital-acquired bacteria, there is no evidence to suggest that it is associated with patient outcomes in this study.

### 3.5 Identifying additional infections in the lab

After identifying UPϕ viruses in other hosts, we returned to the lab to test additional strains. Though only twenty-eight *C. koseri* genomes were available in GenBank, nearly one-third carried a UPϕ virus. In two cases, these *C. koseri* phages were identical to prophage sequences found in urine *E. coli*s. Given this high frequency of infection and similarity to UPϕ901, we tested three unsequenced *C. koseri* isolates (UMB1389, UMB7451, UMB8248) using UPϕ901 PCR primers. The PCR results identified an integrated phage in UMB7451, but it was not actively produced, in contrast to the infected *E. coli* isolates (Supplementary Fig. S5).

We also attempted to establish new infections using filtrate from UMB0901 cultures. Filamentous phages typically rely on conjugative pili as a primary receptor for infection (Mai-Prochnow et al. 2015), and each *E. coli* carrying UPϕ901 in our lab collection also harbored an IncI conjugative plasmid. We identified three uninfected *E. coli* strains (UMB0731, UMB6655, and UMB6890) as candidates for testing UPϕ901’s ability to establish new infections. These strains all clustered together with UPϕ901-infected strains (UMB1220, UMB1526, UMB5814) in a previous gene presence–absence analysis of strains in our collection of urinary *E.coli*s (Garretto 2019), contained an IncI plasmid, and were not already infected by UPϕ901.

Despite their potential as candidate hosts, UMB0901 filtrates that were confirmed to contain the phage did not produce plaques on any of these strains. We were also unable to observe plaques on *E. coli* JE-1, an IncI-bearing strain that is the standard host for UPϕ901’s closest known relative, I2-2 (Bradley, Coetzee, and Hedges 1983). Last, we used PCR to test for phage integration in these candidate hosts but were again unable to confirm new infections.

## 4. Discussion

We have introduced a new filamentous phage, UPϕ901, found in multiple environments, with the largest clade found predominantly in urine *E. coli*s. In addition to infecting *E. coli*, related viruses can be found in *S. enterica*, *K. pneumoniae*, *C. koseri*, and *Y. enterocolitica*. In several cases, identical phage genomes were found in multiple host genera. These phages all share two putative genes of unknown function. We have tentatively named this collection of phages carrying these genes as ‘UPϕ viruses’. Three additional novel ORFs were found in UPϕ901 and fifty-five other UPϕ viruses, and most UPϕ prophages lacking these genes harbored other ORFs in these positions.

These UPϕ viruses are related to phages IKe and I2-2 in the *Lineavirus* genus of *Inoviridae*, but IKe and I2-2 cannot integrate into the host genome and lack the accessory genes that appear to be unique to UPϕ phages (Fig. 2). Given the prevalence of *dif* site integration among filamentous phages and their high sequence similarity to much of the UPϕ901 genome, it is likely that IKe and I2-2 evolved from an integrating ancestor.

Recent work has called into question the current taxonomy of filamentous phages and has suggested that the *Inoviridae* may require substantial revision into multiple new families of viruses (Roux et al. 2019). We are, therefore, hesitant to claim that UPϕ viruses deserve to be identified as a new phage genus. For the time being, we propose that the UPϕ viruses should be considered members of the existing *Lineavirus* genus, with the current members (IKe and I2-2) forming a subgenus of phages that have lost the *dif* site and nearby accessory genes. We acknowledge, though, that this taxonomy could change as the *Inoviridae* are revised and may be complicated by recombination between co-infecting phages.

### 4.1 Relationship to CUSϕ and Ypfϕ

Prior work identified the filamentous prophages CUSϕ and Ypfϕ in strains of extra-intestinal *E. coli* (ExPEC) and *Y*. *pestis* (Gonzalez et al. 2002; Derbise et al. 2007). While these phages have variable accessory gene regions (the same regions where UPϕ has its own novel genes), CUSϕ and Ypfϕ otherwise share 99 per cent nucleotide identity with each other (Derbise et al. 2007). These phages are found in similar hosts as UPϕ viruses and rely on *dif* site integration. In these prior studies, the authors demonstrated both the potential for these phages to increase the virulence of ExPEC (Gonzalez et al. 2002), and also the likely association between Ypfϕ and the modern strains of *Y. pestis* pv *orientalis* associated with the third plague pandemic (Derbise et al. 2007).

As part of the exploration of UPϕ, we compared the individual genes from our UPϕ901 reference sequence to the Ypfϕ prophage region in its type host, *Y*. *pestis* CO92. We found that only genes *II* and *V* have significant homology (Fig. 2), whereas the rest of UPϕ901 is more closely related to phages I2-2 and IKe. Genes *II* and *V* are both involved in regulating phage DNA replication, and this modular split in homology between the disparate viruses likely reflects a deeper history of recombination among the phages.

Because they infect related hosts and might have a history of recombination, we also checked for any instances of co-infection between UPϕ and Ypfϕ in our data. We found one such case (GCA_001519645), corresponding to *E. coli* bacteremia in a patient with sepsis. This assembly, unfortunately, consisted of 206 contigs, and Ypfϕ appears to be split across at least four different contigs. Notably, Ypfϕ is not included on a large contig containing both *dif* and UPϕ, suggesting this Ypfϕ prophage integrated at a different locus using another mechanism or is present as a plasmid in this host. SRA BLAST (Supplementary Fig. S6) confirmed that each phage is fully covered by the reads. It is unknown if the prophage genomes interact. Sachs and Bull (2005) demonstrated that co-infecting filamentous phages (in their case, f1 and IKe) could coevolve to package their genomes together using shared coat proteins. It is feasible that UPϕ and Ypfϕ could also produce chimaeric virions. In fact, the UPϕ region in GCA_001519645 has an early stop codon in its gene *III* and would likely be unviable unless it is able to incorporate Ypfϕ’s version of the attachment protein during assembly.

### 4.2 Tandem repeats and prophage diversity

*dif* site integration typically results in tandem duplication of the phage genome (Mai-Prochnow et al. 2015). It has been observed in CTXϕ and Ypfϕ that hosts harboring a *dif*-integrated phage can be super-infected by other such viruses, with the new phage potentially supplanting the original (Midonet et al. 2019) or integrating in the same region (Derbise and Carniel 2014). In the latter cases, the phage can integrate between the original copies or downstream (Chouikha et al. 2010). It is, therefore, possible to observe tandem triples or even quadruples of inovirus prophages at the *dif* site of infected bacteria, and these downstream homologs are not always identical. Furthermore, this series of integration events can promote hybridization between co-infecting inoviruses when new phages are produced by infected hosts (Davis and Waldor 2000; Derbise and Carniel 2014), as well as co-packaging of phages into shared coats as described above (Sachs and Bull 2005). In the case of UPϕ prophages, we did not always know the exact number of tandem repeats within each host genome, as most sequencing efforts relied on only short Illumina reads that were not resolved into tandem copies during assembly by the original data providers. For UMB0901, we used depth of coverage to infer that UPϕ901 is integrated as a tandem triple.

Given the propensity for tandem prophage recombination, there is additional uncertainty in interpreting and comparing the genomes of UPϕ viruses that presents an additional challenge for taxonomy. The *E. coli* strain ECONIH2 (GenBank: CP014667) was originally sequenced with both Illumina and PacBio technologies and offers a valuable case study. ECONIH2 contains a tandem triple of UPϕ prophages. The first two copies of the phage are identical by nucleotide sequence and contain each of the genes also found in UPϕ901, as well as three additional ORFs. The third copy of the phage in ECONIH2, however, contains only the first of these additional genes followed by a fourth novel ORF. This one bacterial strain demonstrates the potential heterogeneity among co-infecting prophages.

### 4.3 Future work

Additional work remains to understand the prevalence and role of UPϕ viruses in different microbial communities. First, future research will need to characterize the two novel genes, *h1* and *h2*. These genes are found in a region of the genome that often includes genes that alter host behavior or virulence (Mai-Prochnow et al. 2015). In the prototypical integrating inovirus, CTXϕ, this region encodes the cholera toxin genes and their regulators (Waldor and Mekalanos 1996). It appears likely that these two new genes interact with one another, but they share no homology to any known gene, and there is little to hint at their possible functions.

While we have identified putative phages from genome assemblies, it was not always possible to predict phage end positions or the number of tandem repeats. These issues make it difficult to assess the pan-genome of UPϕ viruses, as some might carry additional accessory genes not found in UPϕ901. Future research that includes long-read resequencing of infected hosts and phages released into culture medium would help to resolve these ambiguities.

We also attempted to determine the host requirements for establishing new infections of UPϕ901. We were able to confirm by PCR that new virions are released into the culture medium by infected bacteria, but we were unsuccessful in our attempts to infect new hosts. Our failure to observe new infections may be the result of lacking appropriate host strains for testing or due to low efficiency of establishing new infections. At the same time, UPϕ901’s closest relatives, I2-2 and IKe, each infect *E. coli* harboring IncI conjugative plasmids, and each *E. coli* strain infected by UPϕ901 in our collection also contained an IncI plasmid. It, therefore, appears likely that at least some of these phages rely on IncI-encoded pili, and future work with additional strains and different growth conditions will test this possibility.

It is also possible that different UPϕ viruses rely on different conjugative pili to initiate new infections. The phage attachment protein, g3p, showed the greatest variation across strains, and each of these variants might correspond to different host specificity. Most of this variation was observed in a glycine-rich repeat region that links N- and C-terminal domains of g3p. Previous work showed that this region can diversify in phage IKe in a few generations in the lab, but no effects on phage fitness were observed (Bruno and Bradbury 1997). Those experiments, however, did not test for changes in host range.

## 5. Conclusion

UPϕ viruses represent an exciting new group of filamentous phages. Many inoviruses play important roles in modifying bacterial pathogens within eukaryotic hosts. It remains to be seen if UPϕ viruses, though frequently found as prophages in bacteria from urine, affect the frequency of UTI or other aspects of urinary health. In the case of pre-term infant microbiomes, we observed no correlation between UPϕ prophage presence and patient outcomes. It is possible that UPϕ phages affect bacterial ecology within the urinary environment without changing bacterial virulence. Perhaps more important will be understanding how frequently the phages shift hosts within different environments and whether these new infections alter the phenotypes of multiple genera within a community.

## Data availability

Data from this work is available at figshare (https://figshare.com/s/235048b35ae0617dac68).

## Supplementary Material

veaa030_Supplementary_Data
