# Supplementary material for: UPΦ phages, a new group of filamentous phages found in several members of Enterobacteriales
Source: Virus Evol. 2020 Jun 22;6(1):veaa030. doi: 10.1093/ve/veaa030 (PMC7307601; doi:10.1093/ve/veaa030)
Supplement: veaa030_Supplementary_Data [file veaa030_supplementary_data.zip › Supplemental Figure 1.pdf]

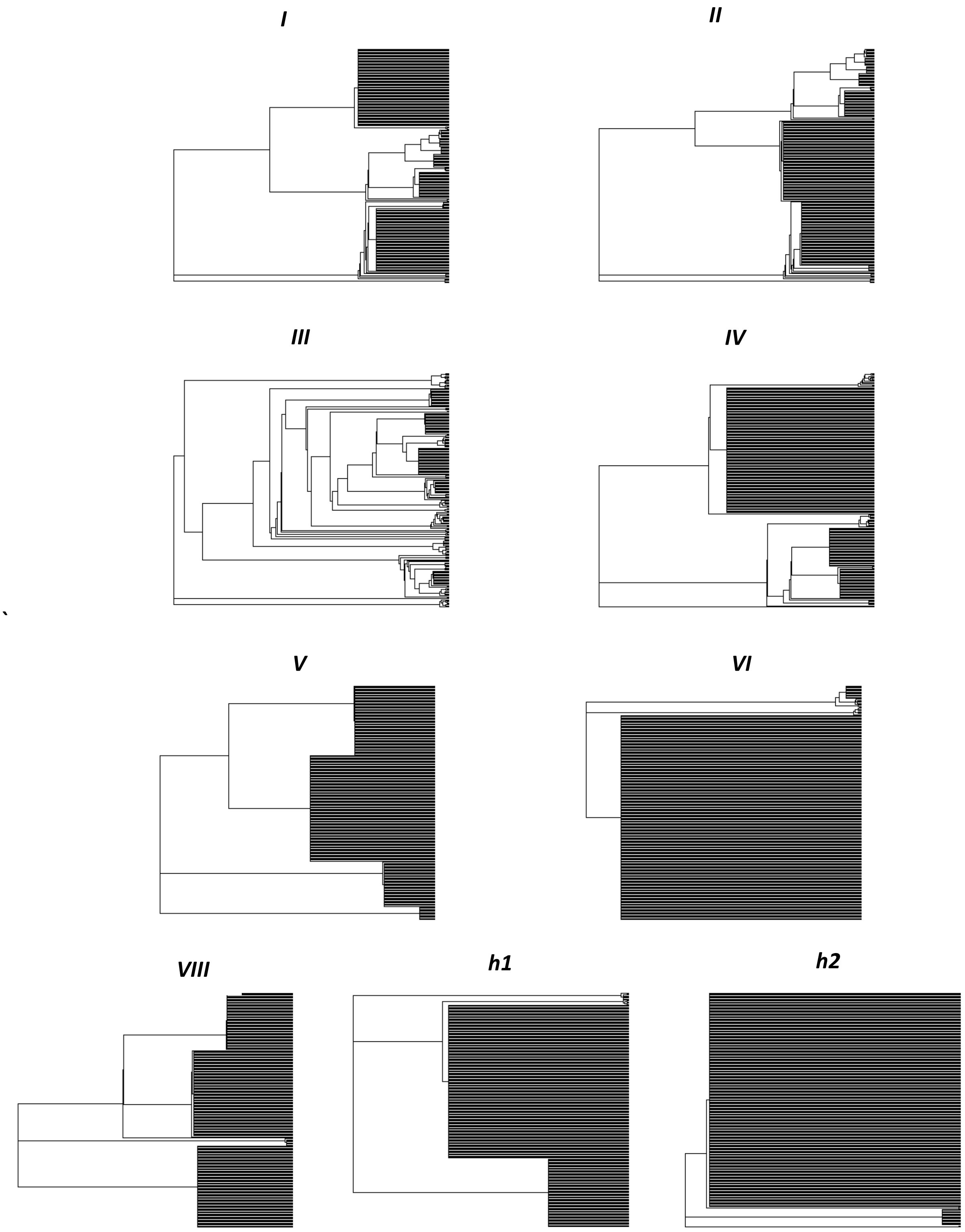

Supplemental Figure 1. UPϕ gene trees. Each tree made from MAFFT alignments and estimated using FastTree with visualizations using ape package in R. Branch lengths ignored to make the extent of polytomies more visible. Each tree labeled by the corresponding phage gene number.
