# Supplementary material for: UPΦ phages, a new group of filamentous phages found in several members of Enterobacteriales
Source: Virus Evol. 2020 Jun 22;6(1):veaa030. doi: 10.1093/ve/veaa030 (PMC7307601; doi:10.1093/ve/veaa030)
Supplement: veaa030_Supplementary_Data [file veaa030_supplementary_data.zip › Supplemental Figure 2.pdf]

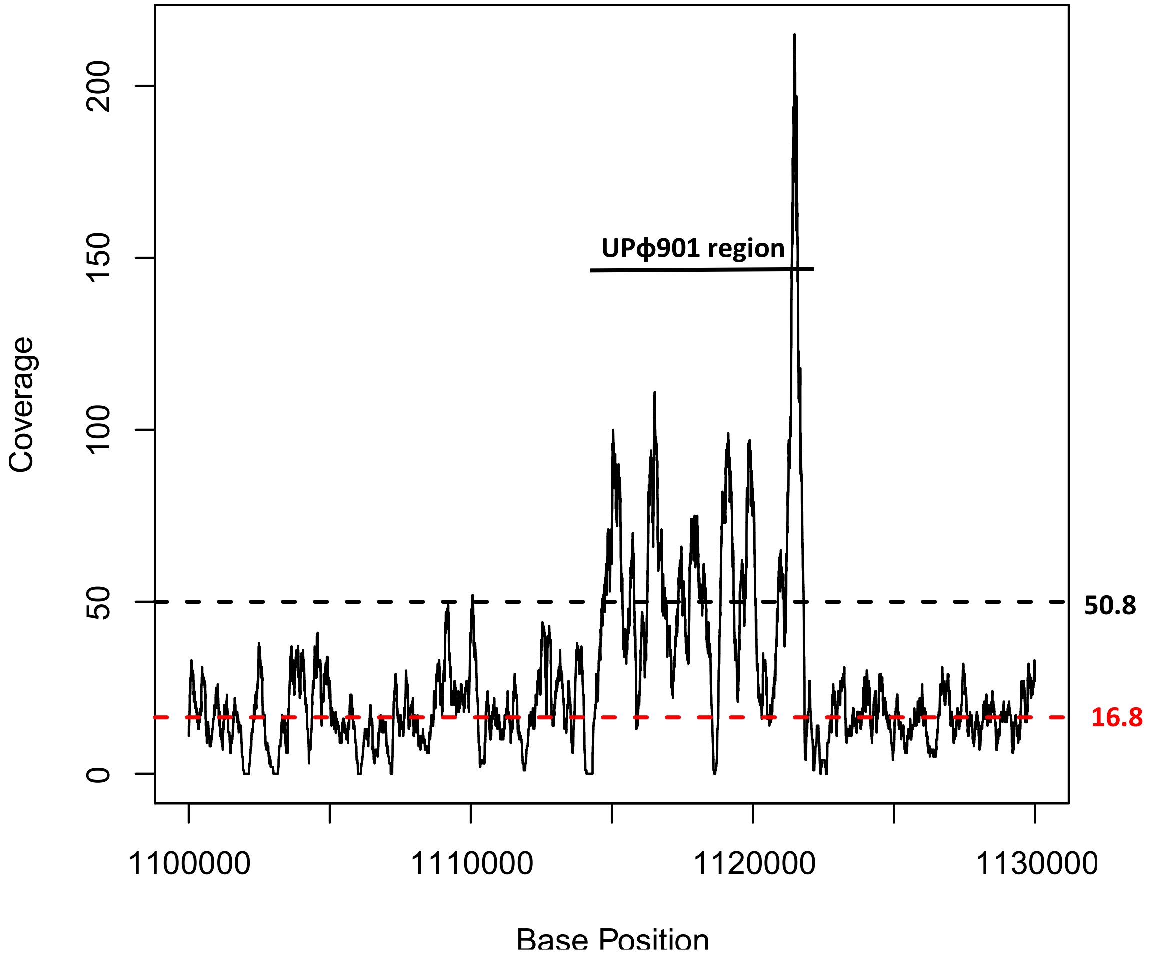


**Supplemental Figure 2. UMB0901 Read Coverage.** The solid black line shows the coverage of the original reads (from ERR1045836) across positions 1100000-1130000 on the first contig in the UMB0901 assembly (GCA_002861225). The red dashed line at 16.8 is the mean coverage in the region outside the UPϕ901 prophage (1100000-1110000; 1125000-1130000) and the black dashed line at 50.8 is the mean coverage in the region containing the prophage (1150000-1123000). The ratio implies the phage is present as a tandem triple.
