# Supplementary material for: UPΦ phages, a new group of filamentous phages found in several members of Enterobacteriales
Source: Virus Evol. 2020 Jun 22;6(1):veaa030. doi: 10.1093/ve/veaa030 (PMC7307601; doi:10.1093/ve/veaa030)
Supplement: veaa030_Supplementary_Data [file veaa030_supplementary_data.zip › Supplemental Figure 3.pdf]

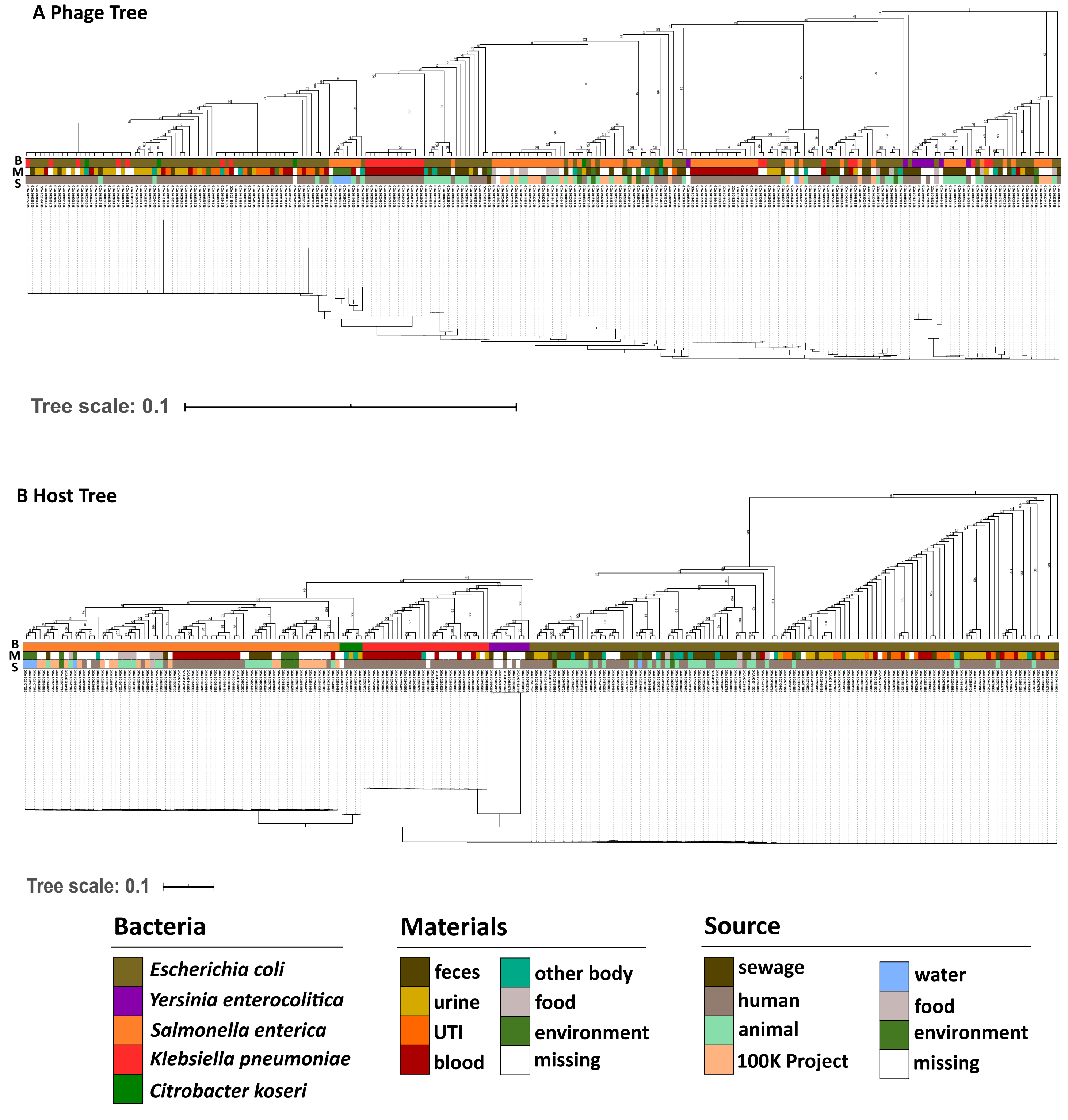

Supplemental Figure 3. High Resolution Phylogenies. Full resolution version of the (A) phage and (B)host phylogenies. Bootstrap supports are shown in the cladogram above the metadata bars, and a phylogeny preserving branch lengths is shown below.
