# Supplementary material for: UPΦ phages, a new group of filamentous phages found in several members of Enterobacteriales
Source: Virus Evol. 2020 Jun 22;6(1):veaa030. doi: 10.1093/ve/veaa030 (PMC7307601; doi:10.1093/ve/veaa030)
Supplement: veaa030_Supplementary_Data [file veaa030_supplementary_data.zip › Supplemental Figure 4.pdf]

A

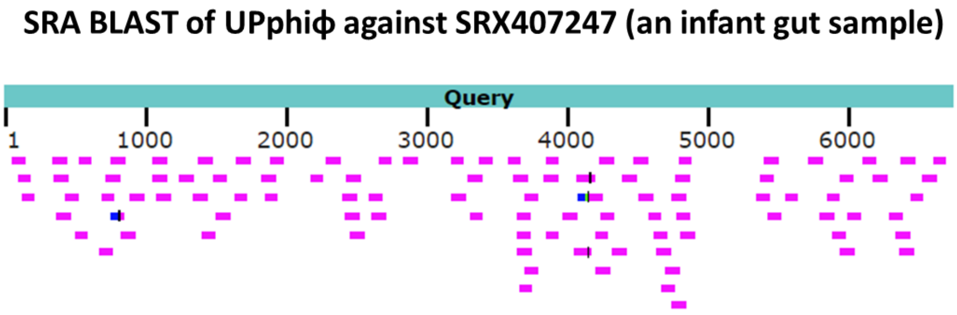

B

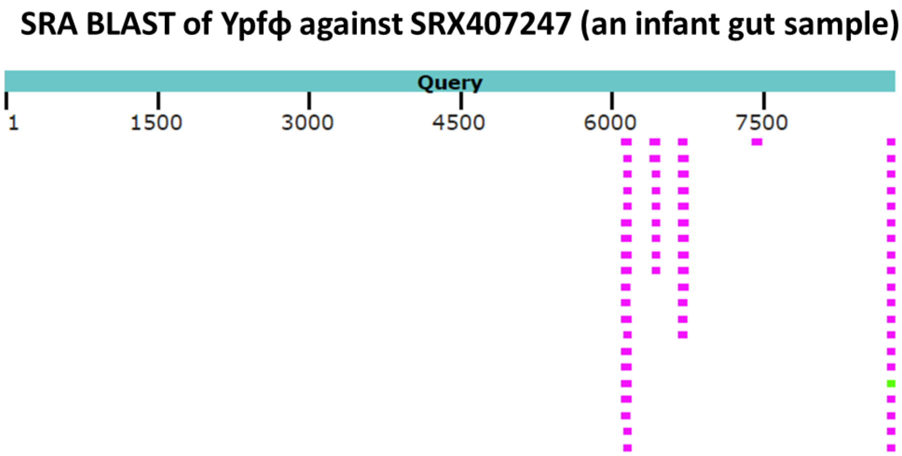

Supplemental Figure 4. SRA BLAST examples. Example SRA BLAST results aligning reads from samples containing UPϕ901 to a (A) UPϕ901 reference or (B) to a Ypfϕ reference. The region hit in the latter contains the two genes shared with over 85% amino acid identity between the phages.
