# Supplementary material for: UPΦ phages, a new group of filamentous phages found in several members of Enterobacteriales
Source: Virus Evol. 2020 Jun 22;6(1):veaa030. doi: 10.1093/ve/veaa030 (PMC7307601; doi:10.1093/ve/veaa030)
Supplement: veaa030_Supplementary_Data [file veaa030_supplementary_data.zip › Supplemental Figure 5.pdf]

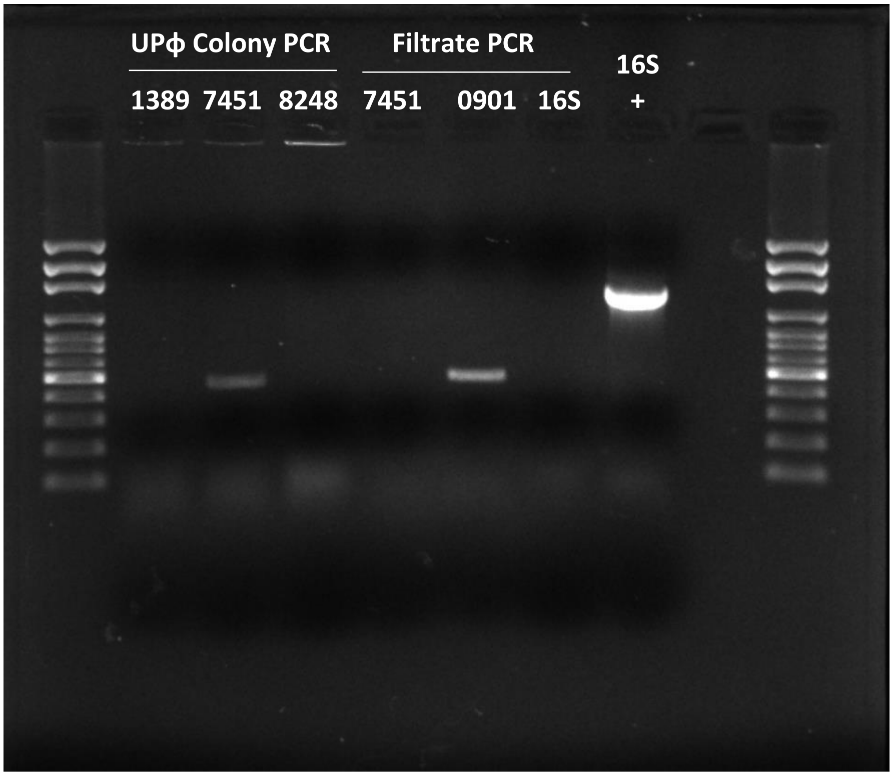

Supplemental Figure 5. ***C. koseri*** **PCR results.** The first three lanes show colony PCR results for 3 *C. koseri* strains, indicating that UMB7451 harbors a UPϕ virus. The next three lanes show PCR results after DNAse treatment to remove bacterial DNA. These show that UPϕ is not secreted by UMB7451 and includes controls for secreted phage PCR from UMB0901 and bacterial DNA depletion. The “16S +” lane is a positive control for the 16S PCR using a UMB0901 colony.
