# Supplementary material for: UPΦ phages, a new group of filamentous phages found in several members of Enterobacteriales
Source: Virus Evol. 2020 Jun 22;6(1):veaa030. doi: 10.1093/ve/veaa030 (PMC7307601; doi:10.1093/ve/veaa030)
Supplement: veaa030_Supplementary_Data [file veaa030_supplementary_data.zip › Supplemental Figure 6.pdf]

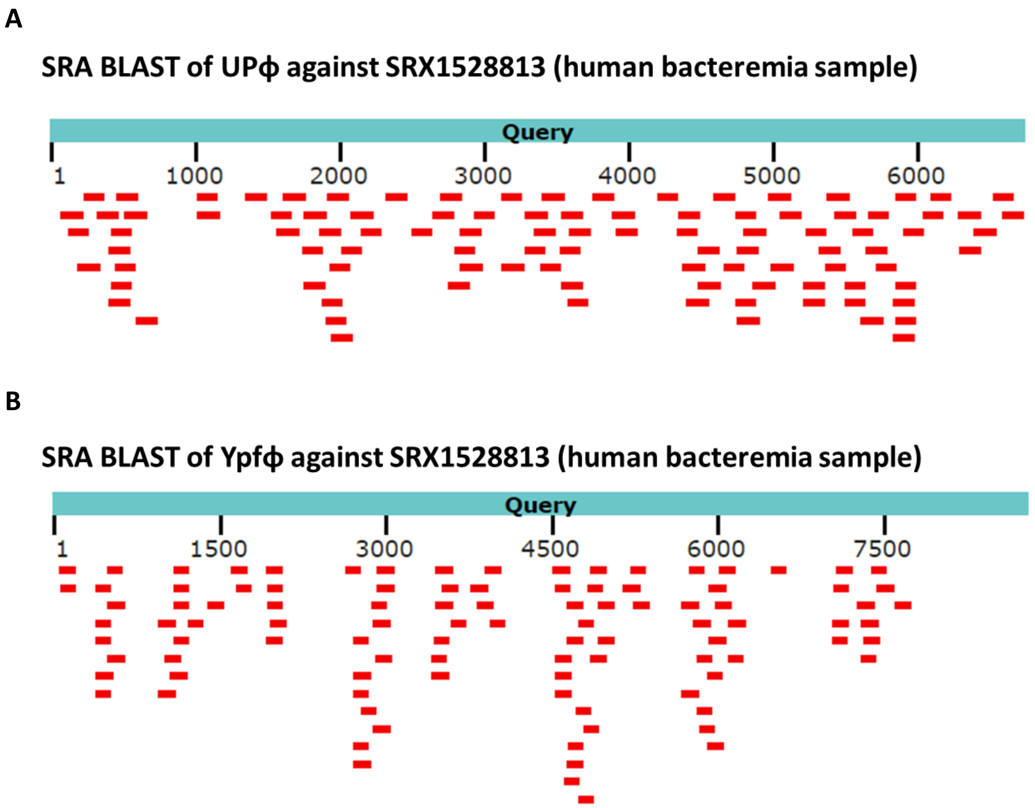

Supplemental Figure 6. GCA_001519645 SRA BLAST. SRA BLAST results aligning reads from GCA_001519645 to both (A) UPϕ901 and (B) Ypfϕ. The results confirm that each phage is present in this genome.
