# Supplementary material for: UPΦ phages, a new group of filamentous phages found in several members of Enterobacteriales
Source: Virus Evol. 2020 Jun 22;6(1):veaa030. doi: 10.1093/ve/veaa030 (PMC7307601; doi:10.1093/ve/veaa030)
Supplement: veaa030_Supplementary_Data [file veaa030_supplementary_data.zip › UPphi_Supplemental Methods.docx]

**Supplemental Methods:** **Identifying true positives in searchsra results.**

Supplement to: UPφ phages, a new group of filamentous phages found in several members of *Enterobacteriales*

Jason W. Shapiro and Catherine Putonti

searchsra (Levi et al 2018) is a valuable tool for scanning a large collection of metagenomes for potential hits to a genome of interest. Because it returns .bam files for any result with an aligned read (using bowtie2), the initial results may contain many false positives. These can be filtered out by first removing any files that correspond to only a handful of aligned reads. In our experience, the easiest way to do this initial step is with a simple file size heuristic. Larger files correspond to more aligned reads, and removing any files smaller than 1 kilobyte quickly brings the remainder down to a manageable number for deeper analysis. In the UPɸ901 searchsra results, this reduced the number of potential hits from over 100,000 to 4329.

We next determined the coverage depth at every locus for our reference genome (here to UPɸ901) using the pileup.sh function of BBmap (Bushnell 2014). It is tempting to rely solely on the mean coverage or the query coverage percent (portion of loci with at least one read aligned), but this can still produce erroneous results. With a short sequence like UPɸ901 (under 10kb), a large metagenome may have many reads with weak alignment across different loci in the reference, leading to an erratic coverage plot that covers the entire genome with high mean coverage but with large variability. In Figure SM1 below, panel A shows a true positive result from the hit in searchsra results to the original UMB0901 genome (SRA ERR1045836) and panel B shows a false positive result to an uncultivated Porphyromonad (SRA SRR5327315). Aziz et al (2015) identified this potential issue with finding phages in metagenomic data and proposed different measures of coverage variability as a means to improve identification. One recommended measure is kurtosis, which measures the length of a distribution’s tail. Another measure we considered here is the maximum difference in coverage between neighboring loci. We term this “maxdiff.” True positives will necessarily have relatively low differences in coverage between neighboring loci (relative to the overall mean coverage), because similar reads will align to those neighbors in a continuous way. False positives, however, will have maxdiff relative to the mean coverage, because neighboring loci that are covered by reads may be hit by very different sets of reads in the metagenome.

We employed these two measures of variability (kurtosis and maxdiff) by visualizing the coverage data as below (Figure SM2). In panel A, the X axis is the percent query coverage, while the Y axis is the ratio of mean coverage to maxdiff. As clearly seen, this separates the data into clear sets. The most obvious set to consider first is the one defined by the line through the origin. For these data, the ratio of mean coverage to maxdiff is higher than the percent query coverage. We used SRA BLAST (Camacho et al 2009) to test cases from each of the visible sets and found that this top-most set always gave true positive results (i.e. numerous hits with high nucleotide identity across the query, see SM3 for an example). The line below occasionally gave a small number of hits to the query with lower percent identity (see SM3 panel B). We therefore kept all cases where the ratio of mean coverage to maxdiff was greater than percent coverage (minus a buffer value of 0.1 to account for noise in the data).

In SM2 panel B, we also plotted the log product of kurtosis and maxdiff against the log of percent coverage. When points from the analysis in panel A are colored in (shown in red), these almost all correspond to cases where the log product of kurtosis and maxdiff were less than 2.5. We observed four points not colored red and added these to our pool of likely true positives for a total of 257 runs.

Last, we downloaded the SRA metadata report from NCBI and parsed the sample and experiment xml files (where available) using the package xml2 (Wickham et al 2019) in R (R Core Team). Where files were missing or not included in the metadata report (e.g. because sequences were associated with EBI), we entered data manually. The metadata are summarized in Supplemental File 1. For each unique BioProject, we then used SRA BLAST to confirm at least one case of a true positive result in each project. These test cases are indicated in the supplemental file. In total, only five projects returned false positive results (corresponding to only 13 of 257 possible results). In three cases, SRA BLAST produced an error and did not return results; in a handful of other cases, the result was only partial and could not be confirmed fully, likely due to either low read depth (or quality) and potentially due to the presence of a related but different phage in the metagenome.

**References**

Aziz, R.K., Dwivedi, B., Akhter, S., Breitbart, M. and Edwards, R.A., 2015. Multidimensional metrics for estimating phage abundance, distribution, gene density, and sequence coverage in metagenomes. *Frontiers in microbiology*, *6*:381.

Bushnell, B., 2014. *BBMap: a fast, accurate, splice-aware aligner* (No. LBNL-7065E). Lawrence Berkeley National Lab, Berkeley, CA (United States).

Camacho, C., Coulouris, G., Avagyan, V., Ma, N., Papadopoulos, J., Bealer, K. and Madden, T.L., 2009. BLAST+: architecture and applications. *BMC bioinformatics*, *10*(1): 421.

Levi, K., Rynge, M., Abeysinghe, E. and Edwards, R.A., 2018. Searching the sequence read archive using Jetstream and Wrangler. In *Proceedings of the Practice and Experience on Advanced Research Computing*: 50.

R Core Team, 2013. R: A language for and environment for statistical computing.

Wickham, H., Hester, J. and Ooms, J., 2019. xml2: Parse XML. R package version 1.2.2. https://CRAN.R-project.org/package=xml2


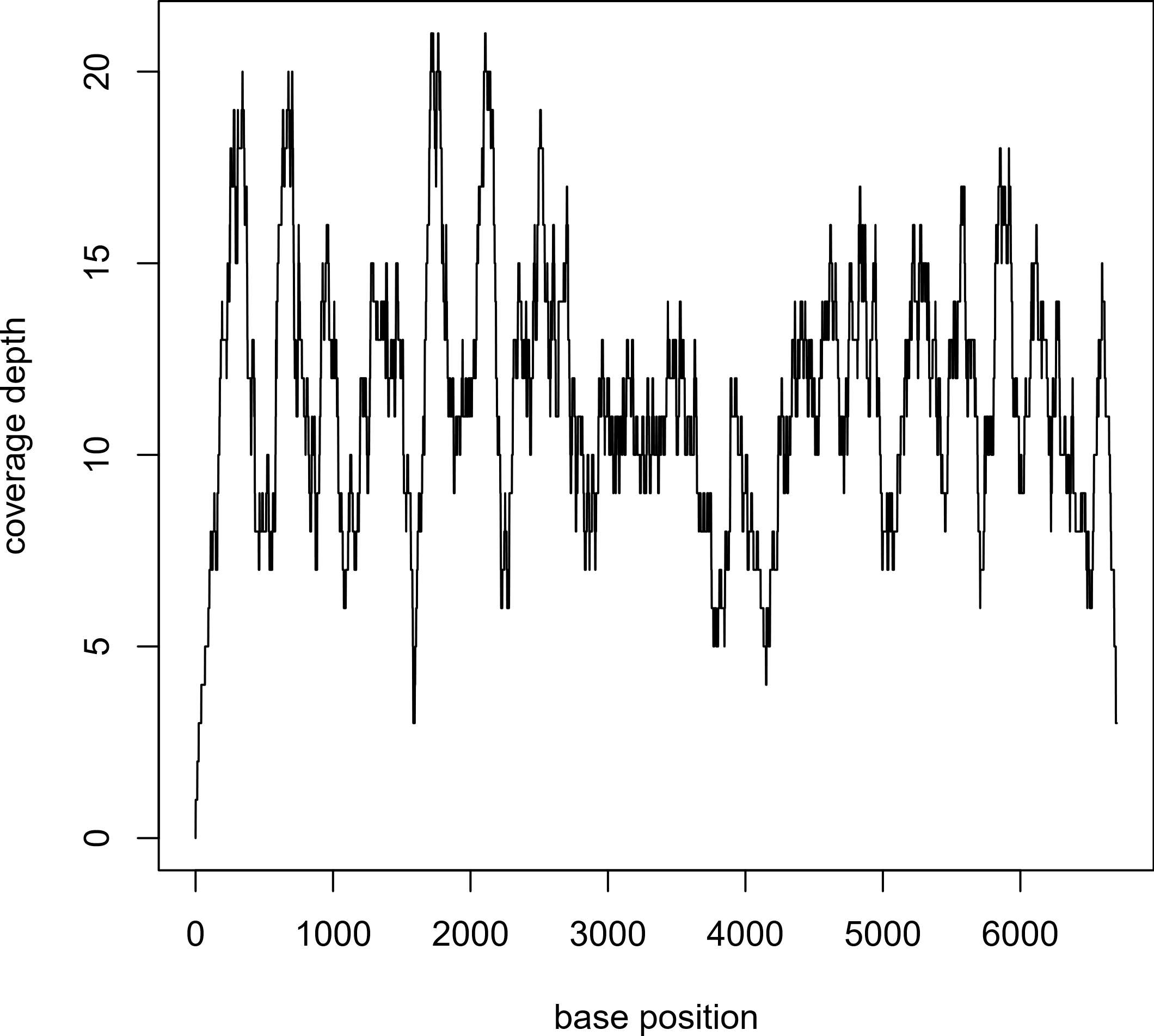


**A**

**B**

**
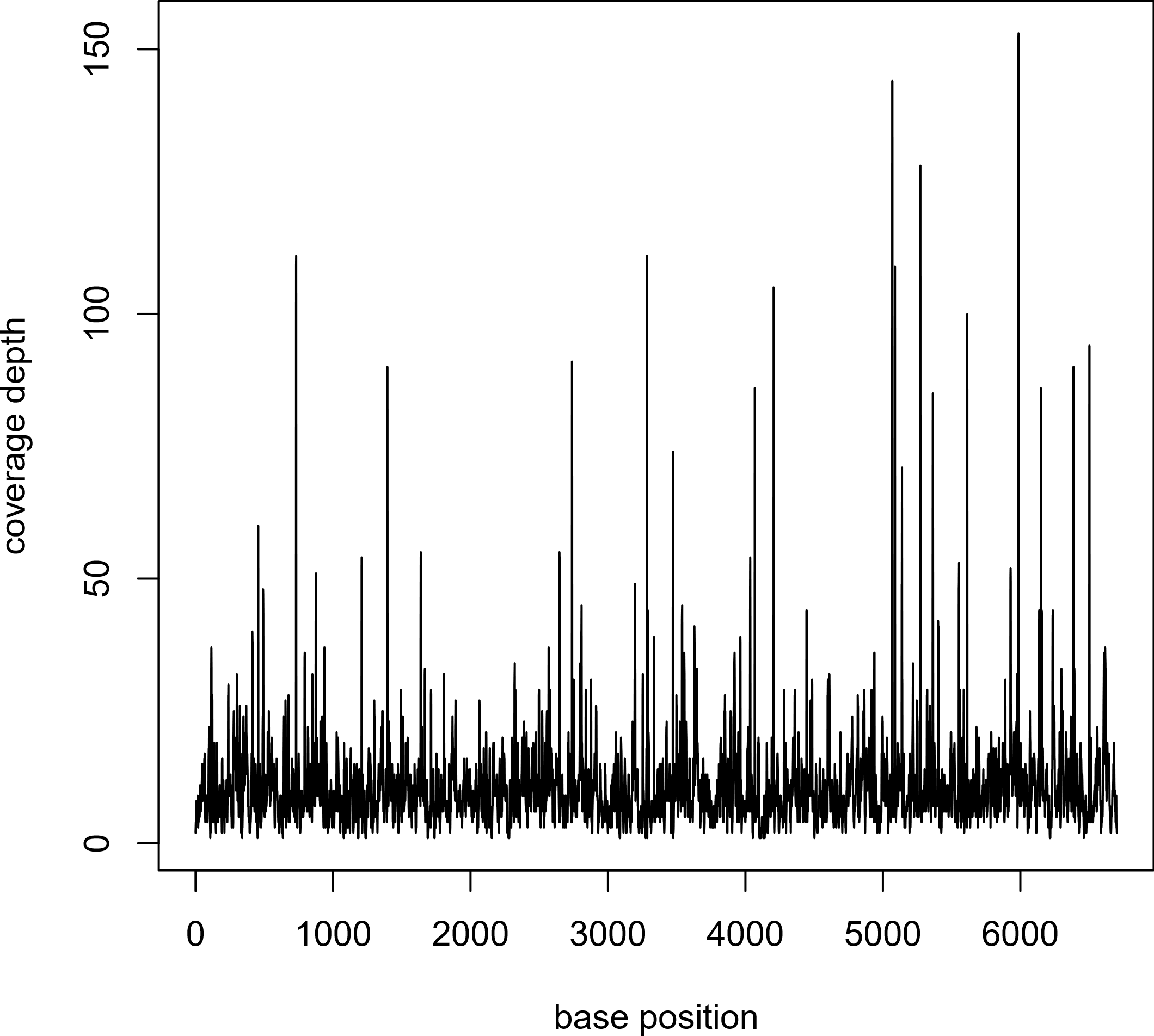
Figure SM1:** Coverage plots comparing SRA runs to UPɸ901 reference genome for (A) a true positive, the original UMB0901 reads (ERR1045836), and (B) a false positive (SRR5327315).

**B**

**A**


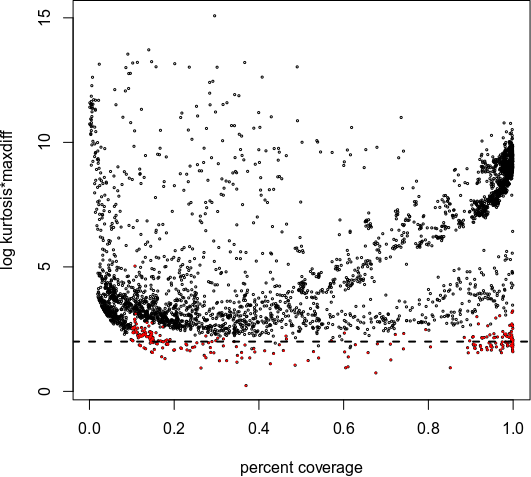

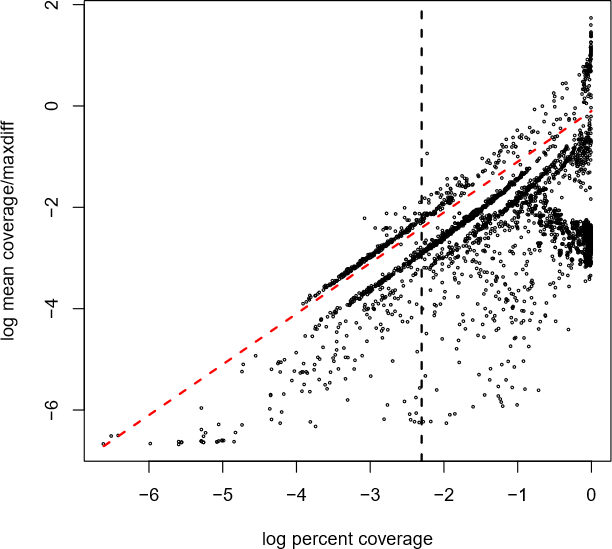


**Figure SM2:** Plots for confirming true positives by measures of coverage evenness. (A) Comparison of the log mean coverage relative to maxdiff against log percent coverage. All points above the red dashed line and right of the black dashed line have over 10% coverage and smooth coverage plots. (B) Comparison of log kurtosis*maxdiff to percent coverage. Red points were identified by the criteria in (A). All points below the dashed line (approximately the average of the red points) were also considered.


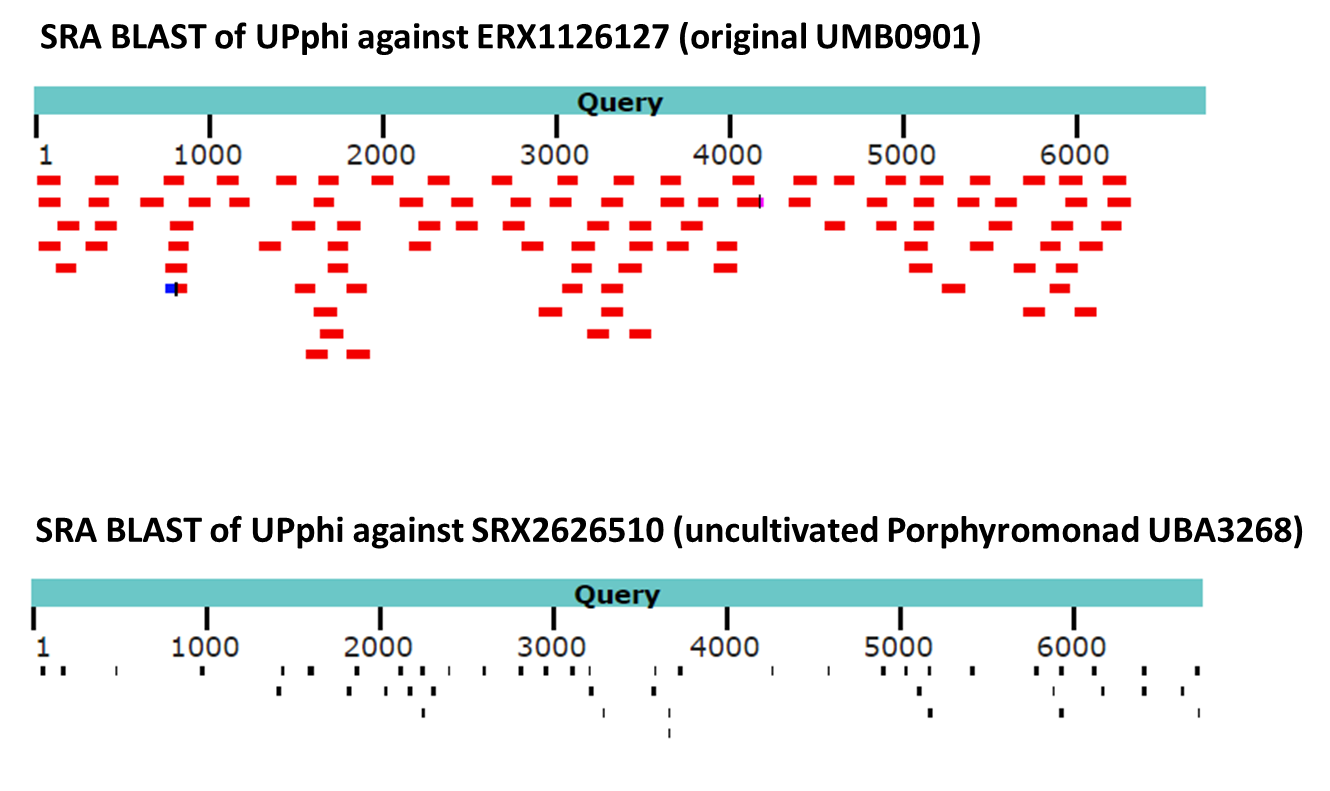


**B**

**A**

**Figure SM3:** SRA BLAST results for a true positive (A) and false positive (B) using the same sequencing data as in Figure SM2.
